# Resolving Molecular Perturbations Near Undercoordinated Metals

**DOI:** 10.1021/acsnano.5c04738

**Published:** 2025-05-22

**Authors:** Alex Poppe, Ishaan Lohia, Margarita Osadchy, Stuart Gibson, Bart de Nijs

**Affiliations:** † School of Physics and Astronomy, 2240 University of Kent, Canterbury CT2 7NH, U.K.; ‡ Physics for Sustainable Chemistry Group, Cavendish Laboratory, 151956 University of Cambridge, Cambridge CB3 0HE, U.K.; § Computer Science Department, 26748 University of Haifa, Haifa 3498838, Israel; ⊥ School of Engineering, Maths and Physics, University of Kent, Canterbury CT2 7NH, U.K.

**Keywords:** plasmonic nanocavity, picocavities, single-molecule SERS, metal nanoparticle, surface-enhanced Raman spectroscopy, machine learning

## Abstract

Metal surfaces can act as efficient heterogeneous catalysts, but their underlying mechanisms are often poorly understood. This is due to the highly transient nature of the underpinning interactions occurring at the single-molecule level, making these difficult to resolve by using traditional analysis techniques. Here, we present a methodology to study metal–molecule interactions near undercoordinated binding sites using single-molecule surface-enhanced Raman spectroscopy (SERS). We demonstrate how machine learning can identify the metal-induced molecular perturbations by recognizing concurrent frequency wandering in vibrational energies, and we compare these peak displacements to extensive DFT modeling to reveal what interactions are occurring. This allows us to resolve how molecules are deformed as they interact with binding sites on metal surfaces. The work provides rare insight into the dynamics and behavior of molecules at catalytically active interfaces and can aid in the rational design of heterogeneous catalysts.

## Graphical abstract

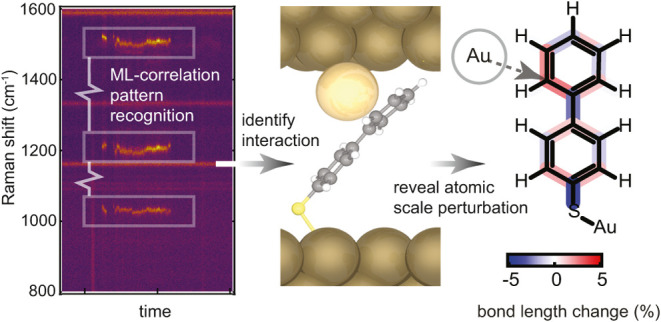

Heterogeneous catalysis has an instrumental role to play in the transition to sustainable operation in both chemical and energy industries.^1^ To this end, there is a need for the rapid development of efficient and selective catalysts, demanding rational design strategies to replace traditional slow serendipitous discovery through trial and error. To address this requirement, fundamental and atomic levels of understanding of the mechanisms involved are required.^2^ The molecule–catalyst interactions underpinning many desired chemical transformations are often complex and by design transient. This makes experimental investigation challenging and computational modeling expensive.^3^ Hence, a need remains for experimental methods that allow direct investigation of metal–molecule interactions, ideally with single-molecule detail.

Undercoordinated atoms on metal surfaces, whether at step-edges, as adatoms or in vertices, are crucial features in heterogeneous catalysis.^4–10^ These provide the essential binding sites and induce perturbation of adsorbed molecules to allow a desirable chemical transformation to occur. In certain geometries, these same atomic-scale features can also provide intense, highly localized field enhancements.^11,12^ As a result, these processes can be directly probed using surface-enhanced Raman spectroscopy (SERS).^11,13^ However, interpreting the resulting vibrational spectra remains challenging as peaks are shifted in frequency and modulated in intensity as the molecules get perturbed.^13–15^ Resolving this would reveal how molecules change their conformations upon binding to catalysts, even before they undergo chemical transformations, providing valuable insights into how catalysts operate and how (by)­products are formed.^13^

To interpret the perturbed Raman spectra, we propose a novel methodology that evaluates frequency wandering in vibrational modes. Evaluating these perturbations decouples interpretation from absolute peak positions and intensities, thereby offering a robust route toward single-molecule SERS (SM-SERS) interpretation.^16^ Perturbations in SERS spectra (i.e., frequency wandering) directly reflect changes to bond strengths and the participating masses. In conjugated systems, this results in correlated/anticorrelated changes as electron densities are redistributed across the molecule.^13^ But to systematically interpret these data, a robust method of extracting correlation behavior from frequency wandering is required. Conventional peak fitting is prone to fitting errors, especially for noisy (high-speed) data. We therefore introduce a machine learning algorithm to effectively identify and label (anti)­correlation patterns in SM-SERS data.

We show how comparing these identified correlations with DFT modeling of perturbed molecules allows identification of the underpinning atomic-scale interactions. We also demonstrate how perturbations play an important role in generating the typically intense and strongly fluctuating SM-SERS peaks. Finally, we reveal how visualization of these correlations provides an effective yet facile tool to visualize how individual molecules interact with catalytically active metal interfaces.

## Results and Discussion

Surface-enhanced Raman spectroscopy (SERS) is used to probe the perturbations of individual molecules as this is one of the few methods offering both the extreme sensitivity and temporal resolution required.^11^ To achieve the necessary field enhancement, the nanoparticle-on-mirror (NPoM) geometry is used.^17^ These structures are realized by forming a self-assembled monolayer (SAM) of molecular spacers (here, biphenyl-4-thiol; BPT) on a flat Au surface. Au nanoparticles are then deposited onto the SAM from a colloidal suspension, resulting in sparsely distributed metal–molecule–metal constructs with intense plasmonic hotspots (illustrated in Figure 1a). These are each identified and positioned for optical interrogation using in-house particle tracking code.^18^ The small spacing between the metal interfaces (∼1.1 nm^19^) allows hotspots to reach field intensity enhancements of |*E* ^2^/*E*_0_^2^| > 10^5^.^20^ This enables SERS spectra to be collected from the ∼200 molecules optically probed in the nanocavity at >27 spectra·s^–1^ (here referred to as *nanocavity spectra)*.^19,21^ In such nanogaps, intense fields cause the stochastic formation of atomic-scale protrusions (adatoms, Figure 1b), which can form on either of the metal interfaces in the gap: “top” or “bottom”.^22–24^ These atomic protrusions support additional intense sub-nm^3^ field localizations termed “*picocavities*” generating strong SM-SERS signals termed *picocavity spectra*.^14,23^ The picocavity spectra are easily recognized in the kinetic scans as a set of transient Raman lines that are shifted, modulated, and dynamic (Figure 1c). The picocavity spectra can reliably be isolated from the nanocavity spectra using a recently developed salient feature extraction method (Figure 1d).^24^ In short, a convolutional autoencoder (CAE) is trained on a large data set of nanocavity spectra (without picocavities). During inference, the model recreates the nanocavity spectra but omits picocavity contributions. Subtracting the reconstructed nanocavity spectra yields the isolated SM-SERS signals (Figure 1c–d).

**Figure 1:**
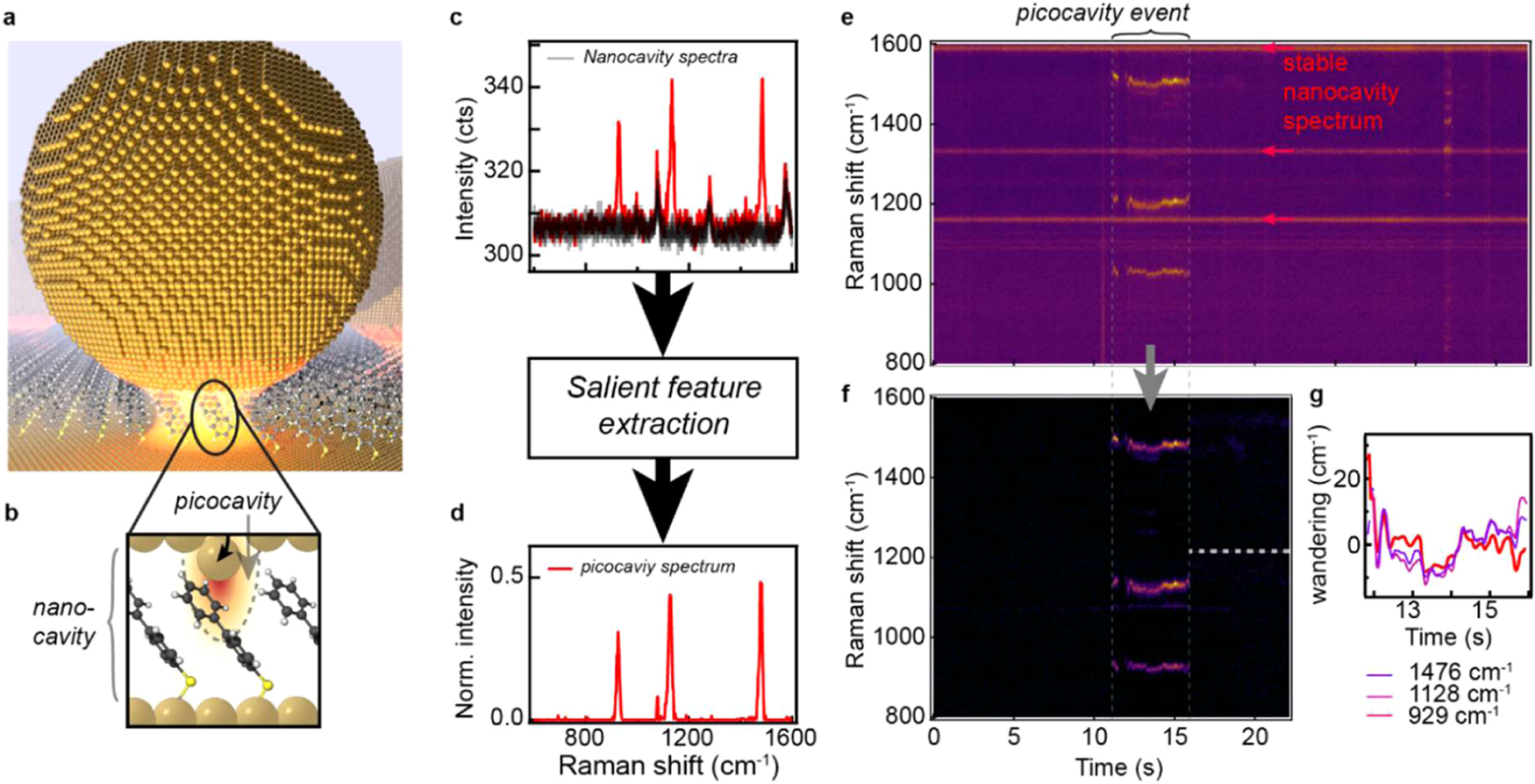
(a) Illustration of a nanoparticle-on-mirror (NPoM) geometry on a self-assembled monolayer (SAM), not to scale. (b) Schematic depicting adatom protruding from lattice generating a *picocavity* to probe nearby single molecules (note: adatoms can occur on either the “top” or “bottom” side). **(c**) Nanocavity SERS spectra of biphenyl-4-thiol (BPT) with a single picocavity spectrum shown in red. (d) Isolated picocavity spectrum. (e) SERS time scan showing stable nanocavity lines and frequency wandering picocavity lines. (f) Isolated picocavity spectra. (g) Graph overlaying picocavity frequency wandering from the mean for three peaks, highlighting the correlated nature.

Since there are no conformational isomers or stable redox states for the BPT used here, we attribute the observed dynamics (frequency wandering) in the picocavity spectra to molecular perturbations arising from interactions with the undercoordinated Au adatom generating the picocavity. We note that modeling a transition from single to bidentate binding for the S-anchoring group does not result in significant perturbations (Figure S1).

Perturbations to molecules result in subtle conformation changes as electron densities and masses are redistributed; this affects multiple vibrational modes as we demonstrate here. This explains why kinetic SM-SERS spectra tend to exhibit strong correlations in their frequency wandering (Figure 1e–g). To study these correlations in frequency wandering in more detail, a large data set of SERS spectra is collected, consisting of >10^6^ SERS spectra from 1400 NPoM geometries in batches of 1000 consecutive kinetic spectra (37 ms cycle time). The resulting data set contains 500 SM-SERS *events* which were isolated using a salient feature extraction method.^24^

### Machine Learning

Previous work has demonstrated that CNNs can successfully extract salient information from empirical Raman spectra.^25^ Siamese-CNNs networks are specifically designed for similarity-based learning and are used here to determine peak–pair relationships (Figure 2a). While our SNN uses a CNN backbone, unlike standalone CNNs, it does not require large, labeled data sets^26^ which would make our analysis framework impractical for application to other molecular structures in future work. This SNN performs a pairwise comparison between concurrent SM-SERS peaks and is able to minimize a distance metric for similar objects and maximize it for distinct objects by using shared weights between each arm of the network. This enables it to identify correlation behavior between peak pairs and assign either positive or negative correlations for overlapping segments in an event (Figure 2b). When correlation changes are detected during an *event*, a new entry is created. For example, if an event is broken down into four segments (100 time steps, 3.7s) and the model predicts correlations of [−, −, +, −], then in total three data points are added, as there are two predicted correlation changes. The resulting correlation matrix (visualized in Figure 2c) reveals predominantly correlated (cyan) events with distinct regions of anticorrelated behavior (magenta) with overlapping points averaged.

**Figure 2:**
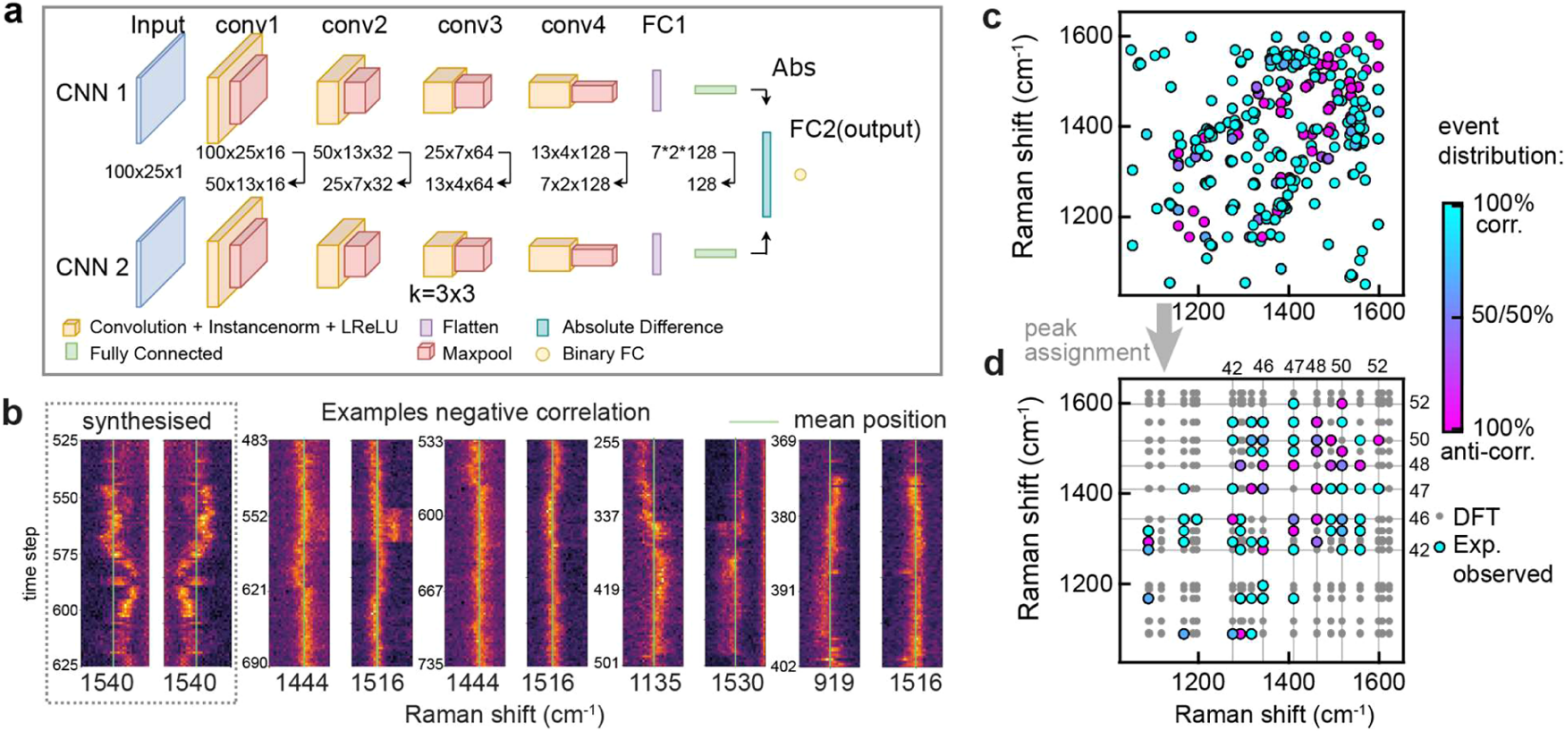
Correlated shifts in the SM-SERS peaks. (a) Siamese-CNN architecture for the pairwise comparison between concurrent peak tracks. (b) Example of synthesized and real anticorrelated peak pairs in picocavity events from which 100-time-step segments are evaluated by the CNN. (c) Correlation matrix showing the degree of correlation for each observed peak pair. (d) Tentatively assigned peak positions (based on frequency ranges reported in ref 24); gray points depict calculated vibrational modes for unperturbed BPT not observed experimentally.

This perturbation matrix provides a fingerprint for all metal–molecule interactions occurring in the probed geometry, likely including both top and bottom picocavities.^23,24^ A tentative peak assignment based on previously reported peak ranges (see ref 24) collapses the data set onto the vibrational modes calculated for the unperturbed molecule (Figure 2d). From this, we can see that most modes behave in a correlated fashion while mode (ν_48_) behaves in an anticorrelated manner. In addition, a couple more anticorrelated points are observed as well: (ν_42_: ν_46_), (ν_45_:ν_47_), (ν_49_: ν_50_), and (ν_50_: ν_52_).

### Modeling

How this interaction gives rise to correlated frequency wandering is modeled using density functional theory (DFT) for a range of possible interaction sites. We postulate that frequency wandering arises from relative motion between the molecule and an undercoordinated adatom. These interactions are approximated by positioning a Au atom at a constrained distance (*d*) from the target interaction site and optimizing the structure. This is repeated for each carbon position and the distance *d* is gradually increased (Figure 3a,b, Figure S2). The energy versus *d* curve reveals that only positions 4′, 2′, 1, and 3 form energetically favorable interactions (Figure 3b, Figure S2). While minima are observed at *d* = 2.2 Å, it is important to note that using a single Au atom overestimates an adatom’s binding potential as in reality adatoms are attached to a Au surface raising the adatom’s coordination number, reducing its binding potential (see Supporting note 1, Figure S3).^6^ Therefore, only Au–molecule interactions for *d* > 2.20Å will be considered.

**Figure 3:**
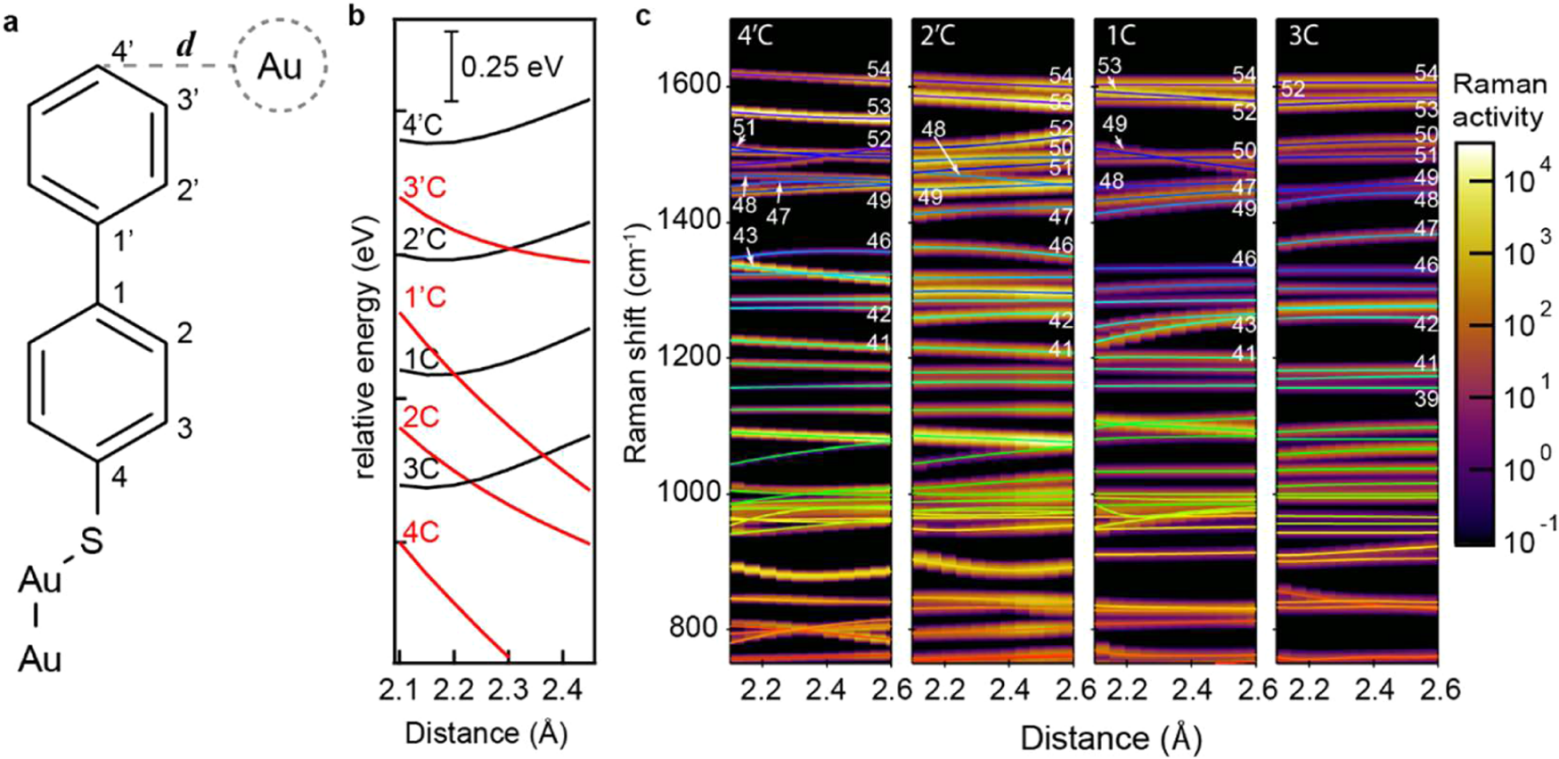
Calculated molecular perturbation by an undercoordinated Au atom. (a) Scheme depicting BPT molecule with potential Au binding sites. (b) Relative energy profile with *d*, normalized to 2.1Å with favorable (black) and unfavorable (red) interactions, plotted with an offset for visibility (see Figure S2 for more details). (c) Corresponding vibrational spectra for each favorable interaction (peaks broadened and shown on a log scale for visibility), fits trace assigned vibrational modes (see Figure S2 for details).

Calculating the corresponding Raman spectra shows distinct frequency shifts with change in *d* for each viable interaction (Figure 3c), with some modes appearing to cross over. We find that by calculating a Spearman rank correlation between mass displacements, we can identify vibrational modes across different geometries (see Supporting note 2, Figure S4). Fitting assigned mode frequencies reveals continuous shifts of varying strengths with a change in *d* with clear correlated trends. Notable exceptions are seen (ν_43_) for 4′C, (ν_48_) for 2′C, and (ν_49_) for 1C, which exhibit anticorrelated behavior, Figure 3c. Figure 3c is plotted on a log scale to visualize weak Raman modes as these can potentially be enhanced by the picocavity’s strong gradient fields.^14,27^ Which modes are enhanced by the strongly inhomogeneous fields depends on the relative position of the adatom and can be calculated from modeled Raman polarizability derivatives using a theoretical treatment introduced by Yao Zhang et al.^27^ In short, the Raman polarizability (α^Raman^) can be defined as a function of the polarizability derivatives $\left(\frac{\delta \alpha^{\mathrm{molecule}}}{\delta \xi^{\left(i\right)}}\right)$ for each atom (*i*) and the respective normalized displacement Φ_ν_^(*i*)^:

$$\alpha_{\nu }^{\mathrm{Raman}}=\sqrt{\frac{\hbar }{{2\mu }_{\nu }\omega_{\nu }}}\cdot \sum_{i=1}^{M}\frac{\delta \alpha^{\mathrm{molecule}}}{\delta \xi^{\left(i\right)}}\phi_{\nu }^{\left(i\right)}$$

where ℏ is Planck’s constant, μ*_ν_* is the reduced mass for mode ν, ω is the frequency, and ξ^(*i*)^ is the coordinate of the atom.^27^ As described by Zhang et al., defining α_ν_^(*i*)^ as the atomistic Raman polarizability for the *i*th atom corresponding to the νth vibration, the atomistic Raman dipole moment ***P***_*i*,ν_^Raman^ for the *i*th atom and νth vibration can then be calculated using the locally experienced electric field ***E***_loc_(***r**_**i**_*).^27^

$$P_{i,\nu }^{\mathrm{Raman}}=\alpha_{\nu }^{\left(i\right)}E^{\mathrm{loc}}\left(r_{i}\right)$$

We then consider:

$$I_{\nu }\propto {\left|\sum_{i=1}^{M}P_{i,\nu }^{\mathrm{Raman}}\right|}^{2}$$

Note that intense optical fields can further influence the molecular structure and intensities in picocavity spectra, but these effects are not included for simplicity.^22,27–29^ Here, only the gradient nearfield and “chemically” perturbed polarizabilities are considered. Plotting α_ν_^Raman^ vs *d* for the perturbed molecules shows how some modes are strongly modulated (see ν_53, 43, 36_; Figure 4a), while others remain mostly unaffected (ν_54_; Figure 4a). Comparing α (for *E*_*z*_) for each interaction shows distinct modulations, and an overall larger increase in polarizability for the two top interactions (Figure 4b, unperturbed molecule shown in black). The respective SERS spectra were then calculated. For this, we consider two cases: the *nanocavity spectrum* where an unperturbed molecule is placed in a flat field polarized in *E*_*z*_ (i.e., across the gap), and for the picocavity where a perturbed molecule is placed in a gradient field *E* ^loc^ calculated according to the analytical model reported in ref 30, see Supporting Note 3 for details (Figure 4c.). Spectra are calculated for each interaction (Figure 4d). For both cases, the molecule is oriented so the BPT backbone is angled θ = 40° from the surface normal reflecting a slightly compressed SAM (Figure 4e) and a small lateral tilt of φ = 30° (how spectra evolve with θ, φ is shown in Figure S5).^31^ For comparison, the nanocavity spectrum is multiplied by the estimated number of molecules optically probed in the nanogap (200), whereas picocavity spectra are plotted for a single molecule.^19^ This shows calculated maximum intensities for picocavities are between ∼100 times higher per molecule than the largest nanocavity peak, agreeing reasonably well with experimentally observed picocavities (Figure 4d, Figure S6). This shows that the surface perturbation, sometimes referred to as chemical enhancement, is important to be included when modeling picocavity spectra.^32^ Without the modulated α^Raman^ modeled picocavity, peaks would be severely underestimated.

**Figure 4:**
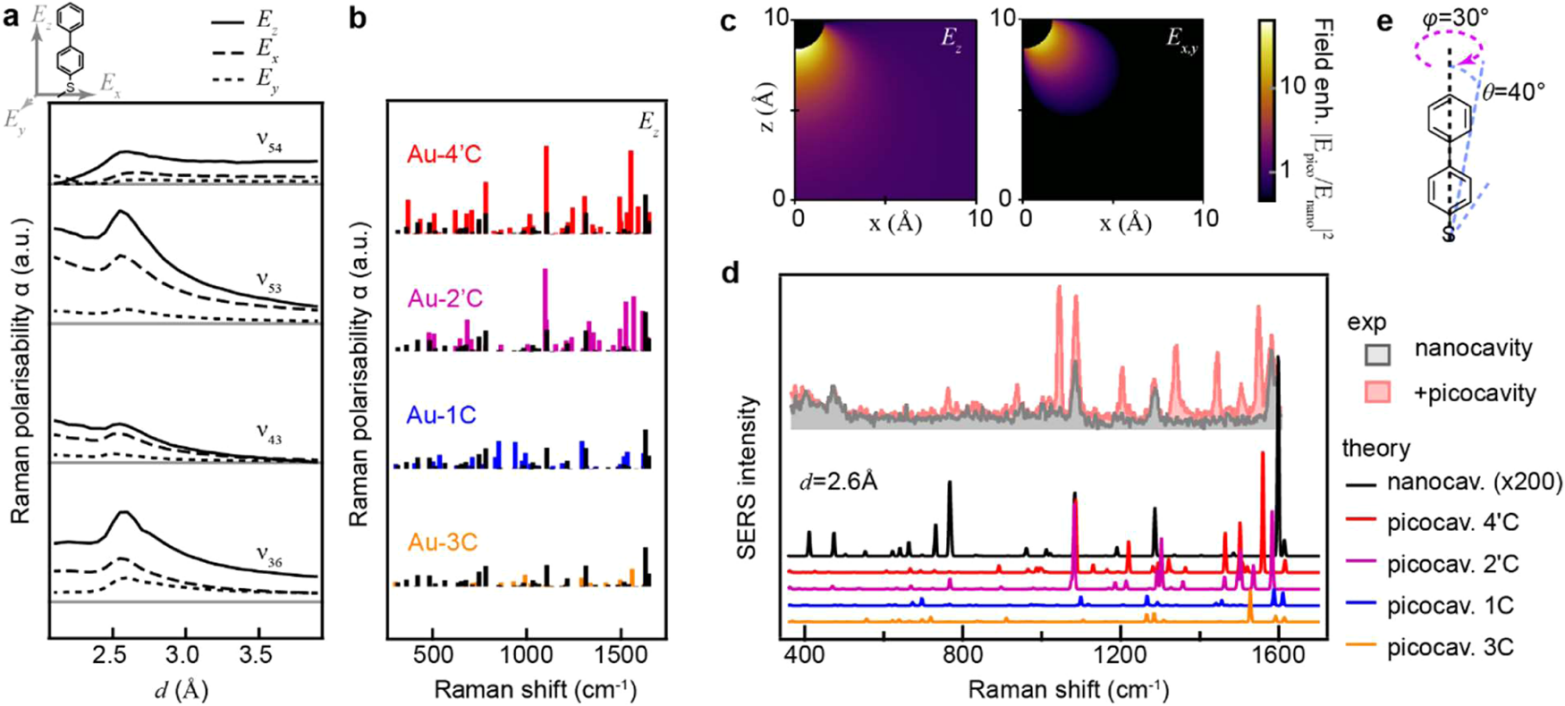
Polarizability and Raman activity of perturbed molecules. **(**a) Polarizability α_ν_^*Raman*^ for Au-4′C interaction plotted for ν_36,43,53,54_ vs *d*. (b) *E*_*z*_ polarizability vs Raman shift for each interaction type with unperturbed BPT shown in black. (c) Analytical description for picocavity field (see Supporting Note 3 and ref 30 for details), for visibility plotted on a log scale. (d) Calculated BPT picocavity spectra and the unperturbed nanocavity spectrum shown in black, broadened for visibility, experimental nanocavity and picocavity spectrum shown in gray and light red, respectively. (e) Tilt angles used for calculating spectra shown in (**d)**; how spectra depend on θ,φ is shown in Figure S5.

Weak picocavity peaks that also do not experience an increase in α^Raman^ are unlikely to be detected; to reflect this, a detection threshold is applied for each interaction type (see Supporting Note 4, Figure S7). For each peak pair, the modeled perturbations (depicted as vectors) can be compared to the experimentally observed correlations (dots, Figure 5a). For the modeling to accurately capture the experimentally observed correlations, represented using magenta (anticorrelated) vs cyan (correlated), should agree with the direction of the vectors (arrows pointing along the diagonal: bottom-left to top-right, are correlated, top-left to bottom-right anticorrelated). The fraction of modeled and measured correlation behaviors that agree can then be determined, reported in bold in Figure 5a. This shows that the experimentally observed perturbations most closely match the modeled Au-2′C interaction with an agreement of 0.92, only ν_49_:ν_50_ disagrees (Figure 5a, while an agreement of 1 is found for 1C, this only represents a single interaction). A scheme of the corresponding 3D structure giving rise to the structure is shown in Figure 5b. This can now be used to determine how the interaction with the metal changes bonds across the molecule based on the DFT modeling. As Figure 5c shows, bonds nearest to the interaction site have become weaker and bonds 1C–1′C and 4C–S gain in double bond character, with a general alternating pattern observed across the conjugated molecule.

**Figure 5:**
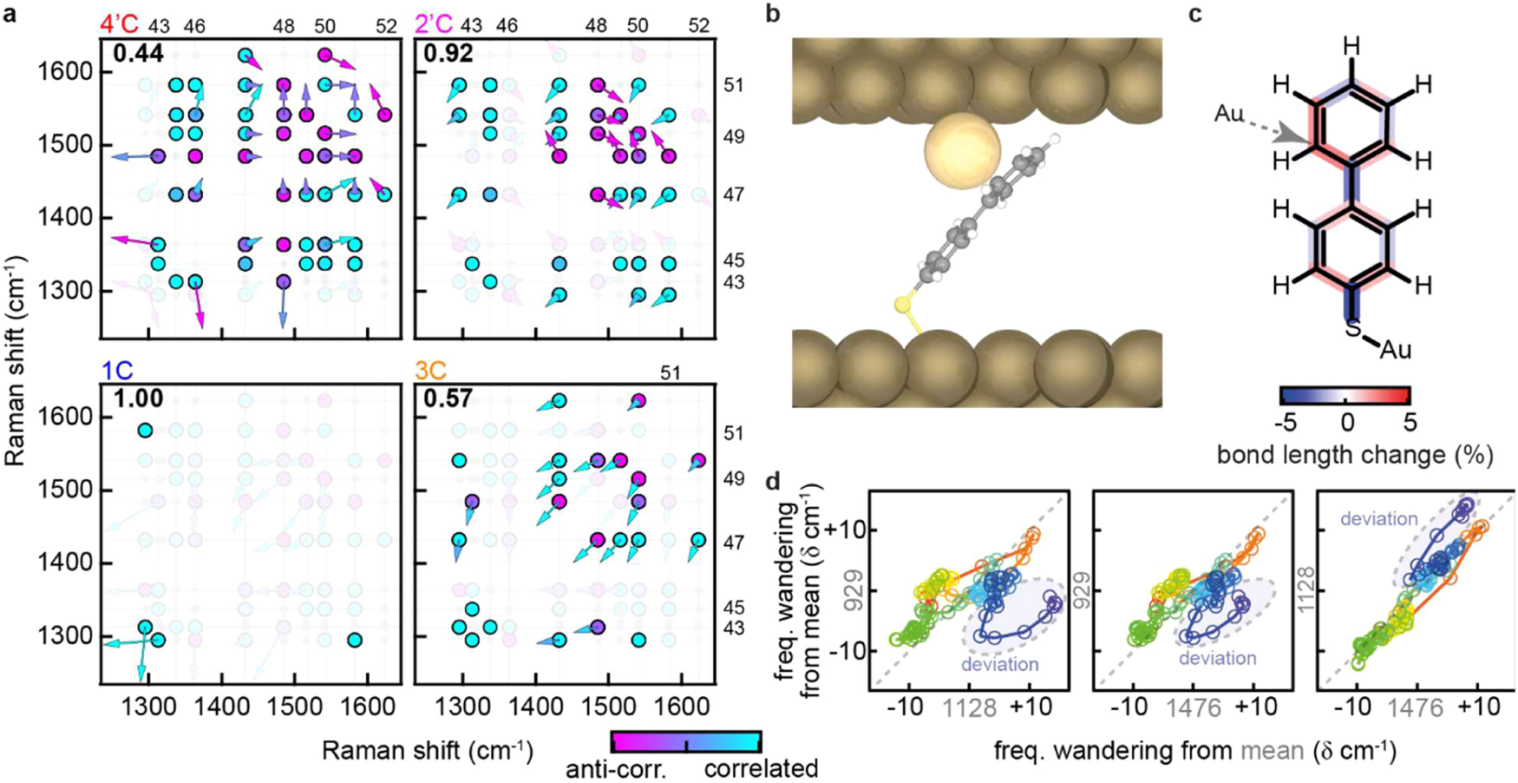
(a) Experimental data for peak pairs shown as circles with their degree of (anti)­correlation visualized using cyan-magenta, depicting correlated to anticorrelated, respectively. Arrows depict the modeled magnitudes in shift for the respective vibrational modes when the metal atom is brought close, again using cyan-magenta for degree of correlation. The fraction of modeled and measured correlations that agree is reported in bold. (b) Geometry of identified metal–molecule interaction (2′C at *d* = 2.6 Å); perturbations further increase with decrease in *d*, see Figure S8 (c) Changes to bond lengths, in %, calculated for the 2′C interaction. (d) Correlation behavior visualized for the event shown in Figure 1e–**g**, showing deviation from correlated changes toward the end of the event (red to blue depicts time).

Finally, we note that the correlation analysis presented does not capture the full granularity of the metal–molecule interactions, as these are unlikely to occur along a single linear axis (e.g., *d*). For example, deviations from the correlation behavior can be readily visualized by plotting the peak positions against each other (Figure 5d). For the event from Figure 1g, a clear deviation from the diagonal (correlated) is observed toward the end of the event (dark blue). This shows that the metal–molecule complex finds a new state which coincides with the end of the picocavity event. This concurrence of deviation and termination of the event is likely related but not observed in all events. For example, SI movie 1 shows the vibrational frequency of three modes plotted as Cartesian coordinates in 3D space. The resulting 3D trajectory represents the metal–molecule complex exploring its energy landscape before returning to its original condition where the event ends. This simple plotting of the correlated peak wandering provides a facile yet effective tool to visualize the behavior of molecules at catalytically active interfaces. These combined findings provide a rare insight into how molecules behave and are perturbed by undercoordinated binding sites on metal surfaces. We believe this methodology to be general and can be applied to other molecules and metal surfaces provided they can generate enough field enhancement for SERS. In addition, thin layers of other transition metals can be layered on Au using, e.g., underpotential or chemical deposition methods, allowing surface chemistries to be tailored toward metal–molecule interactions of interest while retaining the powerful optical properties offered by the noble metals.

## Conclusions

In this work, we demonstrate how correlations between frequency wandering single-molecule SERS (SM-SERS) spectra peaks can be used to resolve interactions occurring between undercoordinated metal atoms and adsorbed molecules. We introduce a machine learning strategy for the systematic identification and labeling of such correlated spectral features. We show how Raman polarizabilities are strongly modulated by the undercoordinated metal atoms, explaining why such strong Raman peaks are observed for single molecules on metal surfaces. We also showcase how the correlated features can be used to track molecules while they explore their energy landscape, showcasing how many more insights can still be obtained from analyzing correlations in frequency wandering of SM-SERS peaks. This now provides a new route toward directly monitoring in real time how molecules behave on catalytically active interfaces and has a strong potential to assist in rational design of new heterogeneous catalysts.

## Methods

All chemicals were ordered from Sigma-Aldrich and used as received. Au nanoparticles were purchased from BBI (OD1, citrate stabilized).

### Sample Preparation

Au substrates were prepared by thermal evaporation deposition of a 100 nm thick layer of Au on a polished silica wafer (roughness < Å). Small (2 × 4 mm) silica pieces were then glued onto the Au using a UV-cured epoxy. After curing, the silica pieces were peeled off, revealing a pristine Au surface. These were submerged in a 0.1 mM solution of BPT in ethanol (200 proof, anhydrous) and left to form a SAM over 18 h. The samples were then rinsed with ethanol and a drop of a colloid Au suspension (BBI, citrate coated, 80 nm) was rested on the sample for 20 s and rinsed off using deionized water. The resulting samples showed a sparse coverage of individual nanoparticle-on-mirror geometries.

### Data Acquisition

All experiments were performed under ambient conditions. SERS data was acquired using a bespoke Raman setup using an in-house particle finding algorithm.^18^ The Raman setup consists of an Olympus BX51 darkfield microscope, a Prior Scientific Proscan III stage for positioning, using a 0.9 NA 100× darkfield objective, and a Lumenera Infinity III camera for imaging. A single-frequency 633 nm diode laser (Integrated Optics) is coupled into the microscope using a 50/50 beamsplitter, and the back-reflected SERS signals are collected using an Andor Shamrock 303i spectrometer with a 600 lines/mm grating and a Newton 970BVF EMCCD (operated in conventional mode). SM-SERS signals are isolated using the previously reported salient feature extraction method.^24^

### Machine Learning

To analyze peak correlations within SM-SERS events, a two-dimensional Siamese-CNN was trained to predict the sign of the correlation between peak pairs, which were processed by the network in the form of overlapping segments of 100-time-step images for a single peak pair, referred to as a *Track*. A Siamese neural network architecture was selected for its ability to minimize a distance metric for similar objects and maximize it for distinct ones using shared weights between each arm of the network, yielding positive and negative correlations, respectively. As, initially, no labeled correlation data was available to train the CNN, synthetic data (augmentation from isolated Tracks) was generated to pretrain the model. This was followed by a fine-tuning process using a subset of real peak pairs whose correlations were manually labeled. As the neural network requires a specific input shape for all images, a minimum duration for a Track was defined to be 100 time steps (3.7s). This selection of Tracks was pooled, and a sliding window was used, with a stride of 5 pixels and horizontally centered on the mean wavenumber of each image, to capture multiple overlapping segments of each Track. The width of the sliding window was allowed to scale to accommodate the full picocavity and was linearly interpolated to a width of 25 pixels in order to fit the shape requirements of the neural network. The CNN arms of the Siamese-CNN contain five hidden layers: four convolutional layers followed by a 128-unit fully connected (FC) layer. A block diagram of the model is shown in Figure 2a. The output FC layers of each CNN arm are combined into one vector by using the absolute difference distance metric between each unit. This combined vector is processed by the “decision head”, a standard FC layer with a single unit. The output of each convolutional layer was normalized using instance normalization, which was initialized from a standardized random uniform distribution, followed by a Leaky ReLU activation function with a slope coefficient, α, of 0.3, and maxpooling with a (2 × 2) stride and kernel size. The model was pretrained for 1000 epochs, using a static learning rate of 0.01 and a batch size of 64 augmented Track pairs. The database of 3850 available Tracks, which could be individually augmented into pairs, underwent a 90/5/5 split which produced a training data set containing 3470 augmented pairs every epoch, one for each Track, as well as validation and testing data sets each containing 190 augmented pairs, which were fixed throughout the training process. Binary cross-entropy loss was used with the Adam optimization algorithm—using parameters β_1_ = 0.9, β_2_ = 0.999, and ϵ = 10^–7^—to adjust model parameters during training. All layers in both the CNN arms and the decision head were regularized using the L2 weight decay with a regularization factor, γ, of 0.1. Clipnorm was used to clip the calculated gradients to the maximum L2-norm value to avoid the problem of exploding gradients. For fine-tuning, the model was trained for an additional 13 epochs with a static learning rate of 0.001, before early stopping was implemented to prevent model overfitting. All other pretraining hyperparameter values were also used in the fine-tuning process. As there were 455 real Track pairs whose correlations were manually labeled, the fine-tuning data set was partitioned using *k*-fold cross-validation strategy, with a *k*-value of 10. Each partition held approximately 410 training samples and 45 testing samples; the partitioning was performed based on each whole Track, meaning that where multiple overlapping image pairs would make up an entire Track, that set of image pairs would remain within the same partition. This was done to avoid creating a testing data set that was too similar to the training pool, thus forming a trivial evaluation task.

### DFT Modeling

The commercial package Gaussian09 was used for DFT modeling of the molecular perturbations and corresponding vibrational spectra. For this, the UB3LYP hybrid density functional was paired with the DEF2TZVP basis set and the D3 dispersion correction with Becke-Johnson damping.^33^ A BPT molecule’s geometry was optimized, and an Au atom was placed near each carbon atom at a constrained distance and the structure was reoptimized at increasing distances using the potential energy surface scan feature as part of the ModRedundant feature included in Gaussian09. This was repeated near each carbon atom position over distances from 2.1 to 3.6 Å at steps of 0.05 Å. Raman spectra, displacements, and polarizability derivatives were then calculated for each geometry. The distance *d* was extended to 3.9 Å for the 4′C interaction to visualize the change in α_ν_^Raman^ with a decrease in *d*.

## Supplementary Material





## Data Availability

Data set S1 contains all experimental data used for the analysis and is available at DOI 10.17863/CAM.113166. The machine learning codes used for this research are available at github.com/nanophotonics/scanalyser.

## Notes

Effect of coordination number (Supporting Note 1); Spearman correlation for peak assignment (Supporting Note 2); analytical model for picocavity (Supporting Note 3); determining detection threshold for modeled picocavity peaks (Supporting Note 4); bidentate binding sulfur group (Figure S1); change in relative energies vs adatom distance for each interaction site (Figure S2); Au-4′C optimized distance vs coordination number (Figure S3); calculated Spearman rank correlations (Figure S4); angle dependence of calculated Raman intensities (Figure S5); examples of various picocavity spectra compared to the calculated picocavity spectra (Figure S6); determining detection thresholds (Figure S7); graph showing the continual C–S bond length change with decreasing (Figure S8) (PDF)
Visualization of how a molecule explores its energy landscape by plotting three wandering peak positions against each other, using the marker size of the spectral evolution indicated in real time (Movie S1) (MP4)
