# Supplementary material for: Resolving Molecular Perturbations Near Undercoordinated Metals
Source: ACS Nano. 2025 May 22;19(21):20120–7. doi: 10.1021/acsnano.5c04738 (PMC12139032; doi:10.1021/acsnano.5c04738)
Supplement: Supplementary file 1 [file nn5c04738_si_001.pdf]

Resolving Molecular Perturbations near Undercoordinated Metals

Supporting Information

Alex Poppe[1], Ishaan Lohia[2], Margarita Osadchy[3], Stuart Gibson[1*], Bart de Nijs[2*]

[1]School of Physics and Astronomy, University of Kent, Canterbury CT2 7NH, U.K.
[2]Physics for Sustainable Chemistry Group, Cavendish Laboratory, University of Cambridge, Cambridge CB3 0HE, U.K.
[3]Computer Science Department, University of Haifa, Haifa 3498838, Israel.
[*]Correspondence can be addressed to Stuart Gibson: s.j.gibson@kent.ac.uk, Bart de Nijs: bd355@cam.ac.uk

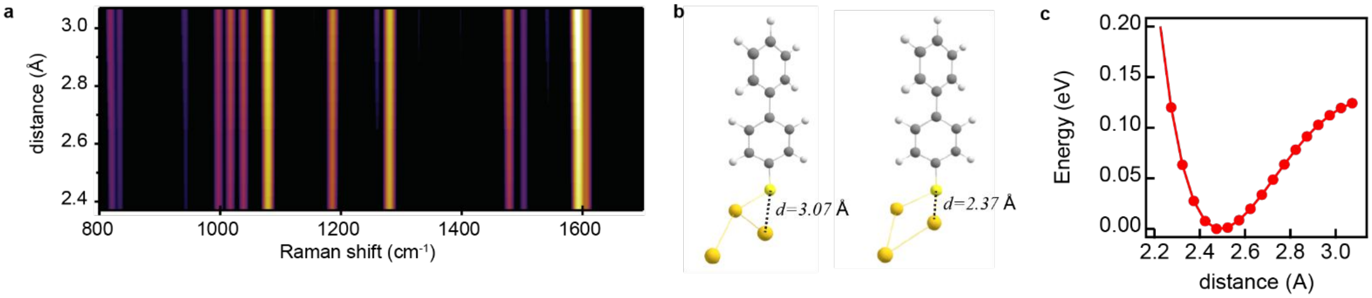

Fig. S1. Bidentate binding sulfur group. Since an S group can bind to more than one Au atom the extent of this on the spectra is modeled. Here vibrational spectra for BPT with are calculated with an additional gold atom placed at constrained distances from the S atom. This shows that no significant changes to the Raman lines are observed suggesting that, whilst this is likely to occur, it is unlikely to cause the observed frequency wandering.

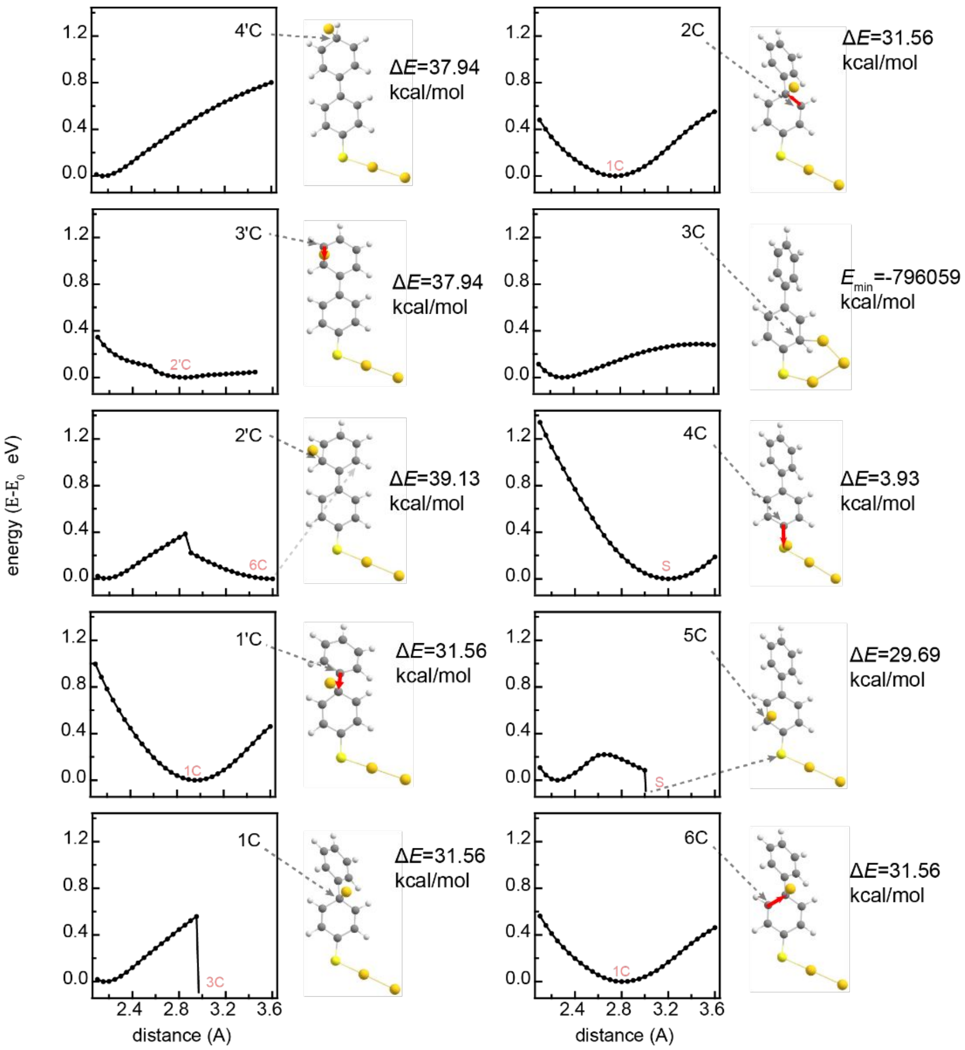

Fig. S2. Change in relative energies vs adatom distance for each interaction site. An Au atom is placed at a constrained distance of 2.1 Å near each of the possible interaction sites on a biphenyl-4-thiol molecule, and the structures are optimized using density functional theory (ub3lyp/def2tzvp, see methods section for more details). The constrained distance is then gradually increased with steps of 0.05Å and geometry optimized. From 4'C (top) across the molecule we find alternatingly favorable/unfavorable binding near the respective interaction sites. As the dictated distance to the targeted interaction site is increased the Au atom finds new binding sites (indicated with red arrow on diagram, labelled in red on trace). A steep drop in energy is observed when the Au atom is able to combine with the S atom or the other Au atoms, which in reality will likely be already occupied by other Au atoms from the surface, not modelled here. For completeness relative energies are also reported in kcal/mol with respect to the lowest energy configuration of the set (i.e. 3C; where the Au adatom combines with the other Au atoms). Though this steep drop in energy for 3C is not representative of the experimental system as in all

cases the Au atom will be bound to a Au surface. For the S group this would depend on whether another atom is bound or not, which as shown in Fig. S1 has little effect on the spectra.

Supplementary Note 1: Effect of coordination number

The coordination number (number of neighbors interacting with the atom, *CN*) of a Au atom effect its binding potential and subsequently its catalytic activity.[1,2] To demonstrate this Au atoms with either 1,3,5 neighboring Au atoms were optimized near the 4’C binding site. Letting the complexes optimize shows how the Au-4’C distance increases with coordination number, see Fig. S4. This is what is expected and aligns with previous modelling by Lopez et al. that shown binding energy is reduced with coordination number.[2] Lopez et al. also shown how at *CN:*9 (i.e. a flat Au FCC surface) no favorable binding occurs for CO or O, which agrees with our previously reported experimental observations for CN-Au binding.[3,4]

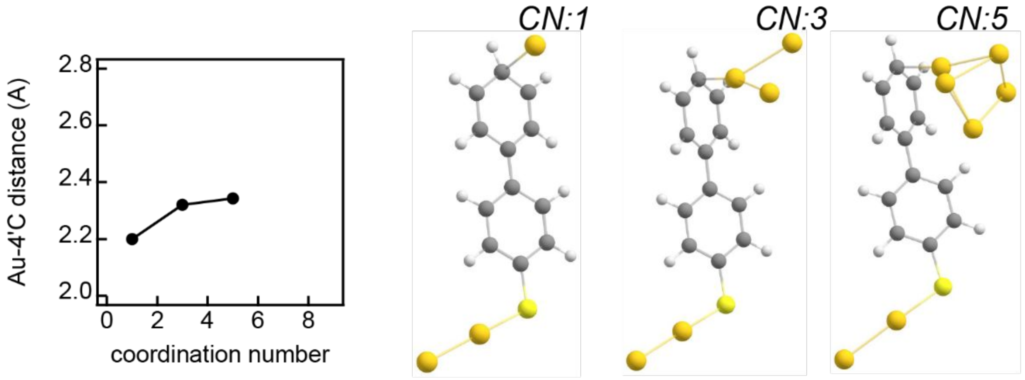

Fig. S3: Au-4’C optimized distance vs coordination number (*CN).*

Supplementary Note 2: Spearman correlation for peak assignment

To allow comparisons between DFT calculations, all final structures (and corresponding properties such as displacements, polarizability derivatives) were rotated such that the 4’C, 1’C, 1C and 4C distances are maximized in y and the distances between the remaining carbons are maximized in x. An array containing displacements for each atom (excluding the Au atoms) is created, concatenate principal axes, and multiplied by the corresponding mass. Spearman rank correlations ($\rho_{vv'}$) are then calculated between a range of vibrational modes from two configurations using:

$$\rho_{vv'} = 1 - \frac{6\sum_{k=1}^{3}\sum_{i=1}((\Phi_{v}^{(i)} - \Phi_{v'}^{(i)})\cdot m^{(i)})^{2}}{n(n^{2}-1)}$$

Where $\Phi_{v}^{(i)}$ is the displacement for each atom (i) in direction (k) for vibrational modes ν and ν' multiplied by the respective atom’s mass $m^{(i)}$. The absolute values are then compared (as vibrations between configurations can have opposite phase).

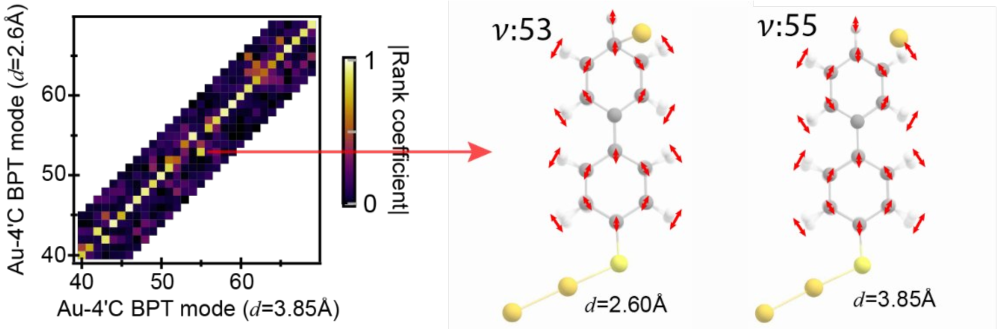

Fig. S4: Calculated Spearman rank correlations between configurations Au-4'C BPT at d =3.85Å and d =2.60 Å showing maxima deviating from the diagonal where modes have crossed.

Supplementary Note 3: Analytical model for picocavity

The analytical model is in the main text for the gradient picocavity fields approximates the atomic scale feature as a metallic half ellipsoid, as proposed by Baumberg[5]. To calculate the wavelength dependent polarizability ($\tilde{\alpha}_j(\lambda)$) of the half ellipsoid we use:

$$\tilde{\alpha}_j(\lambda) = \frac{\alpha_j(\lambda)}{\varepsilon_0 V} = \frac{1}{\left[\frac{\epsilon_m(\lambda)}{\epsilon_g} - 1\right]^{-1} + L_j L_s(\lambda)} \quad , \; j = \{x,y,z\}$$

With V being the volume of the half ellipsoid with semiaxes a_j, Then ϵ_m is the metal's permittivity (here Au, using interpolated data from Johnson and Christy[6]), and ϵ_g the permittivity of the gap medium (here using $\epsilon_g = 1.5$). We consider the z-axis to be perpendicular to the gap and x,y parallel. The L_j term describes the structure parameter accounting for the anisotropy of the ellipsoid with $\sum_{j=1}^{3} L_j$=1.

$$L_z = \begin{Bmatrix} (1-e^2)e^{-3}\left(-e + 0.5\ln\left(\frac{1+e}{1-e}\right)\right), & e^2 = 1 - \varphi^{-2} \;\; if\; a_z > a_{x,y} \\ e^{-2}\left(1 - \varphi\frac{\sin^{-1} e}{e}\right), & e^2 = 1 - \varphi^2 \;\; if\; a_z < a_{x,y} \end{Bmatrix}$$

With $\varphi = a_z/a_{x,y}$, for the case where $a_z = a_{x,y}$ we use $a_z = 1/3$. For the presented modeling we assumed an aspect ratio of φ =2.0.

To account for half embedding of the elliptical asperity in a metal surface a multiplicative structure factor L_s is included[7]:

$$L_s(\lambda) = 1 - \mathcal{N}\frac{\epsilon_m - \epsilon_g}{\epsilon_m + \epsilon_g} \quad , \; with\; \mathcal{N} = 0.19$$

$\mathcal{N}$ is a constant tuned to match the analytical model to the numerical simulations.

Finally, to calculate the picocavity field enhancement we use:

$$\frac{E_{pio}}{E_{nano}} = E_{nano}^{-1}\binom{E_{x,y}}{E_z} = \binom{0}{1} + \frac{1}{\left(1+\frac{\delta}{a}\right)^3}\left[\tilde{\alpha}_z \cos\theta \binom{\sin\theta}{\cos\theta} - \binom{0}{1}\right]$$

With $r = a + \delta$ being the distance outside the metal atom of atomic radius a. For more details on the model see ref [5]

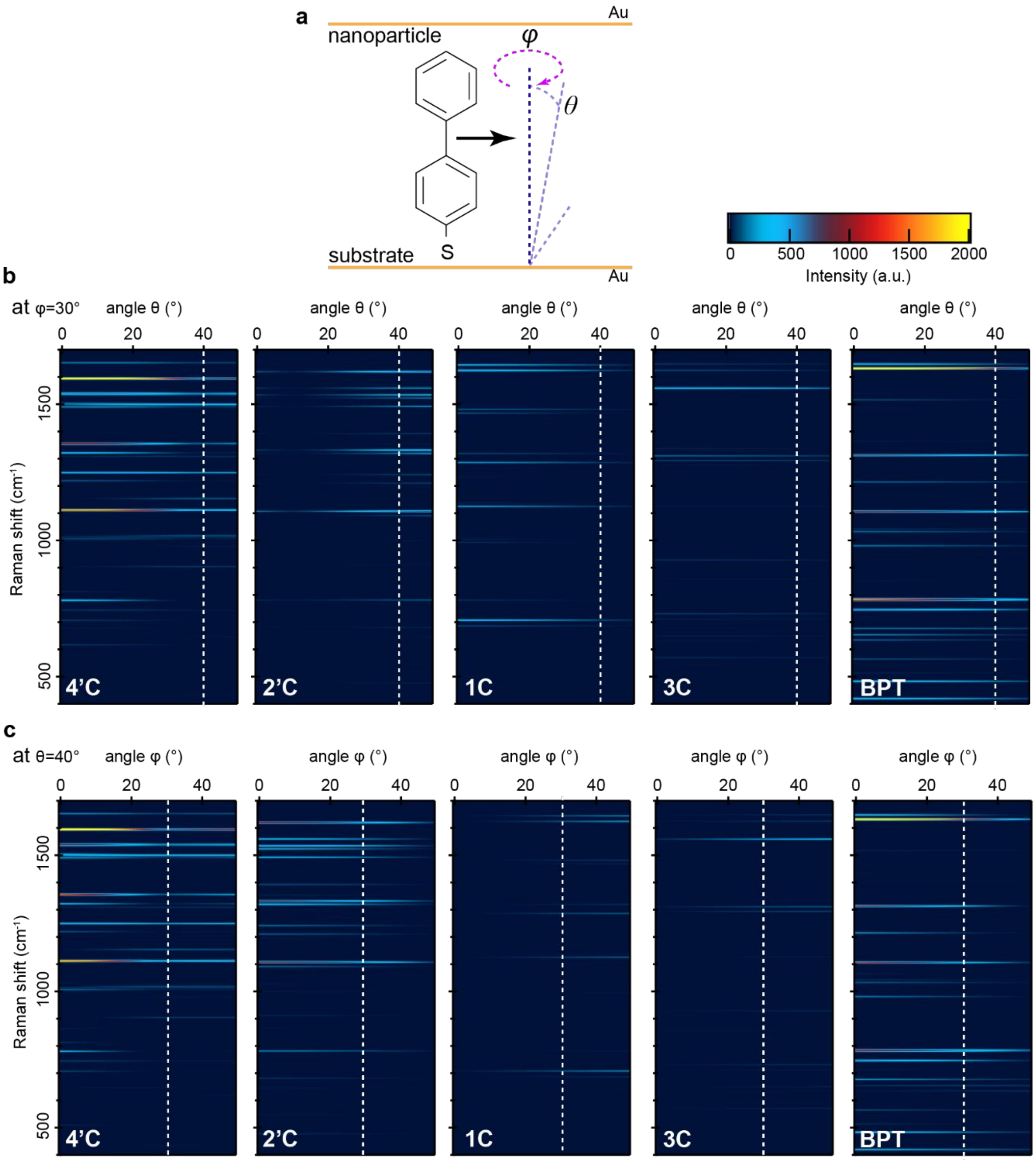

Fig. S5. Angle dependence of Raman intensities. **a**) Schematic depicting the BPT molecule and the respective angles considered. For the nanocavity calculation (unperturbed BPT) a linear polarized field is applied. For the picocavities a gradient field is applied for each polarization as reported in the main text and supplementary note 3. **b**) 2D plots visualizing how calculated intensities evolve

with $\boldsymbol{\theta}$ (calculated for $\boldsymbol{\varphi} = \mathbf{30}°$). **c**) Evolution with $\boldsymbol{\varphi}$, calculated for $\boldsymbol{\theta} = \mathbf{40}°$. Angles used in the main text indicated white dashes lines ($\boldsymbol{\theta} = \mathbf{40}°$, $\boldsymbol{\varphi} = \mathbf{30}°$).

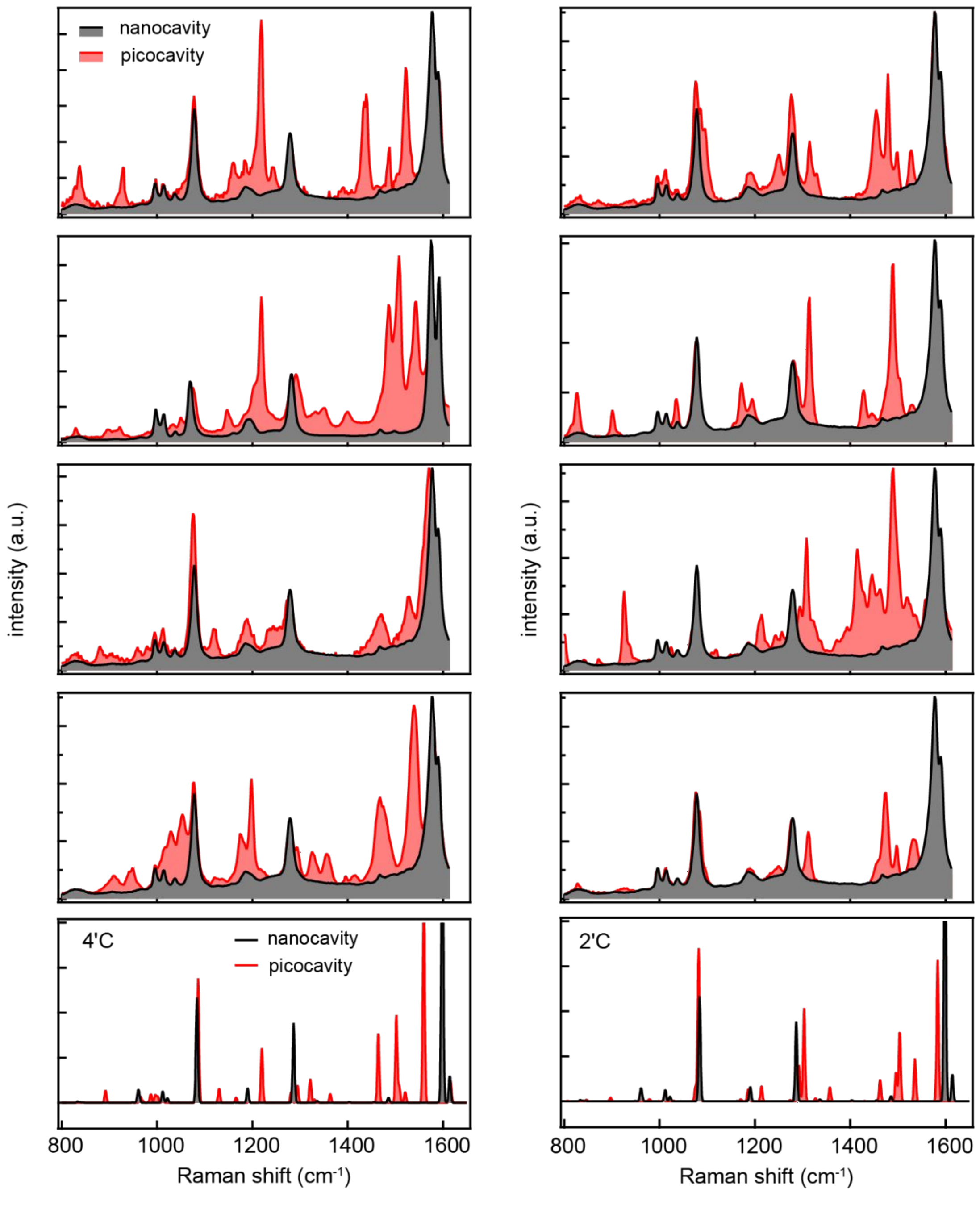

Fig. S6. Examples of various picocavity spectra compared to the calculated picocavity spectra. The calculated spectra can generate similarly shifted and amplified peaks with some tentative agreements in positions. Note the modeled nanocavity is multiplied by 200 whereas the calculated picocavity is for one molecule. Though as discussed in the text, since experimental picocavities are highly variable and dynamic

it is nearly impossible to form a close enough to match an experimental spectrum to simulations, prompting the need for the proposed correlated perturbation analysis.

Supplementary Note 4: Determining detection threshold for modelled picocavity peaks

We use the relative intensities of the nanocavity peaks (experimental vs calculated) to provide an estimate for the detection threshold (DT). The relative standard deviation (RSD) of the background noise is determined in the notch region, yielding RSD=1.2 counts. Since peak intensities (I_ν) are experimentally measured in integers (counts), we set the DT at the equivalent of 2cts (=3.8%) of the amplitude of the most dominant nanocavity peak (at 1575cm^{-1}). We then calculate the peak detection probability P_ν as:

$$P_\nu = \frac{1}{1 + e^{-(I_\nu - DT)}}$$

The combined detection probability is then calculated as:

$$P_{nm} = P_n P_m$$

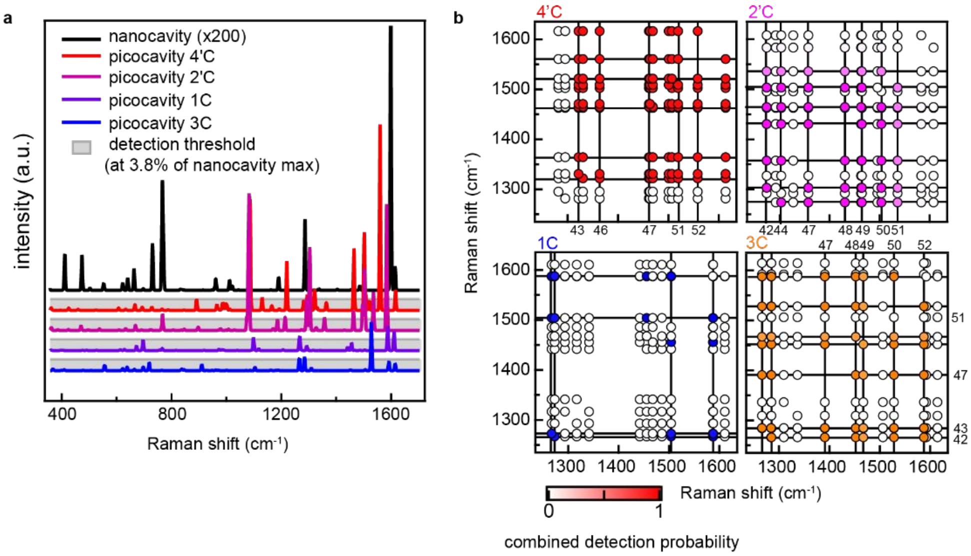

Fig. S7. Determining detection thresholds. **a**) calculated picocavity spectra with detection threshold indicated in grey. **b**) Picocavity peak matrix with combined detection probability depicted in color saturation.

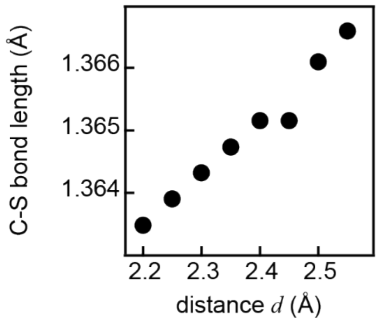

Fig. S8. Graph showing the continual C-S bond length change with decreasing $\boldsymbol{d}$ (shown here for the 2'C interaction). As noted in the main text, the magnitude of the bond length change will differ from real perturbations as this is poorly captured in modelling. The direction of change however is expected to be correct as discussed.

Movie S1.

Fitted peak positions from a single picocavity event for three wandering peaks plotted as cartesian coordinates. The colors indicate time and each datapoint represents the results from a single spectrum. The size of the datapoints is used to emphasize time, showing how the metal-molecule complex explores its energy landscape and, in this case, returns to its initial configuration towards the end of the event.

Data S1. (separate file)

Dataset containing the raw BPT data used is available at:
doi.org/10.17863/CAM.113166

References

(1) Fujita, T.; Guan, P.; McKenna, K.; Lang, X.; Hirata, A.; Zhang, L.; Tokunaga, T.; Arai, S.; Yamamoto, Y.; Tanaka, N.; Ishikawa, Y.; Asao, N.; Yamamoto, Y.; Erlebacher, J.; Chen, M. Atomic Origins of the High Catalytic Activity of Nanoporous Gold. *Nature Mater* **2012**, *11* (9), 775–780. https://doi.org/10.1038/nmat3391.

(2) Lopez, N.; Janssens, T. V. W.; Clausen, B. S.; Xu, Y.; Mavrikakis, M.; Bligaard, T.; Nørskov, J. K. On the Origin of the Catalytic Activity of Gold Nanoparticles for Low-Temperature CO Oxidation. *Journal of Catalysis* **2004**, *223* (1), 232–235. https://doi.org/10.1016/j.jcat.2004.01.001.

(3) Carnegie, C.; Griffiths, J.; de Nijs, B.; Readman, C.; Chikkaraddy, R.; Deacon, W. M.; Zhang, Y.; Szabó, I.; Rosta, E.; Aizpurua, J.; Baumberg, J. J. Room-Temperature Optical Picocavities below 1 Nm3 Accessing Single-Atom Geometries. *J. Phys. Chem. Lett.* **2018**, *9* (24), 7146–7151. https://doi.org/10.1021/acs.jpclett.8b03466.

(4) Griffiths, J.; Földes, T.; de Nijs, B.; Chikkaraddy, R.; Wright, D.; Deacon, W. M.; Berta, D.; Readman, C.; Grys, D.-B.; Rosta, E.; Baumberg, J. J. Resolving Sub-Angstrom Ambient Motion through Reconstruction from Vibrational Spectra. *Nat Commun* **2021**, *12* (1), 6759. https://doi.org/10.1038/s41467-021-26898-1.
(5) Baumberg, J. J. Picocavities: A Primer. *Nano Lett.* **2022**, *22* (14), 5859–5865. https://doi.org/10.1021/acs.nanolett.2c01695.
(6) Johnson, P. B.; Christy, R. W. Optical Constants of the Noble Metals. *Phys. Rev. B* **1972**, *6* (12), 4370–4379. https://doi.org/10.1103/PhysRevB.6.4370.
(7) Wind, M. M.; Vlieger, J.; Bedeaux, D. The Polarizability of a Truncated Sphere on a Substrate I. *Physica A: Statistical Mechanics and its Applications* **1987**, *141* (1), 33–57. https://doi.org/10.1016/0378-4371(87)90260-3.
